# A_2_Se_2_C_2_ (A = Na–Cs): crystalline compounds featuring ^−^Se–C≡C–Se^−^ dianions with short-range structural disorder

**DOI:** 10.1039/d6ra01467d

**Published:** 2026-05-05

**Authors:** Tim Mattick, Marc Hetzert, Uwe Ruschewitz

**Affiliations:** a Department of Chemistry Greinstraße 6 50939 Köln Germany uwe.ruschewitz@uni-koeln.de +49 221 470 3933 +49 221 470 3285

## Abstract

Crystalline powders of A_2_Se_2_C_2_ (A = Na–Cs) were prepared either by reacting A_2_C_2_ with two equivalents of elemental (grey) selenium in liquid ammonia or by thermally decomposing ASeC_2_H at 200 °C under dynamic vacuum. IR and Raman spectra unambiguously confirm the presence of ^−^Se–C≡C–Se^−^ anions. X-ray powder diffraction patterns exhibit only a few very broad reflections, consistent with a primitive cubic arrangement of the alkali metal cations. The anions occupy the interstitial voids between these cations and display local orientational disorder. Only through a combined approach employing Rietveld and pair distribution function (PDF) analyses of synchrotron powder diffraction data, supplemented by density functional theory (DFT) calculations, it was possible to establish a consistent structural model for the anion disorder.

## Abstract 2

The crystal structures of A_2_Se_2_C_2_ (A = Na–Cs) with an unusual local disorder of ^−^Se–C≡C–Se^−^ anions were solved by a combination of conventional IR/Raman and Rietveld analyses, DFT calculations, and total scattering data (pair distribution function).

## Graphical abstract

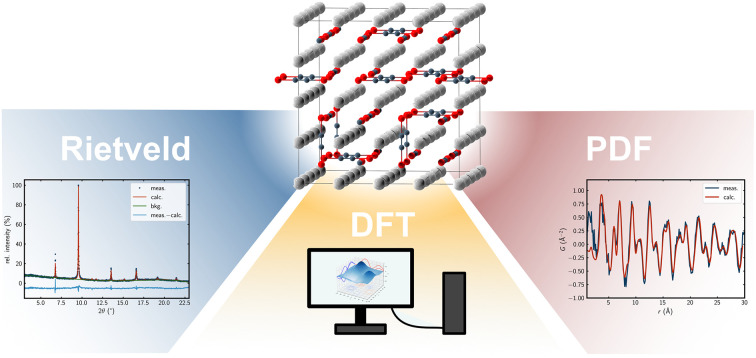

## Introduction

In his pioneering work more than a century ago, Moissan reported on the synthesis of alkali metal acetylides with the composition A_2_C_2_ (A = Li–Cs).^1,2^ Several decades later, based on X-ray powder diffraction data, the crystal structures of these acetylides were investigated, confirming the existence of C_2_^2−^ anions, as postulated by Moissan.^3–5^ Notably, in all these crystal structures, the C_2_^2−^ dumbbells are ordered at ambient conditions. Only at higher temperatures, a rotational disorder of the acetylide anions was observed for Li_2_C_2_ (>770 K),^6^ Na_2_C_2_ (>580 K),^7^ and K_2_C_2_ (>420 K).^7^

In 1963, the first report of a ternary acetylide, namely KAgC_2_, was published.^8^ Its crystal structure revealed the existence of linear 
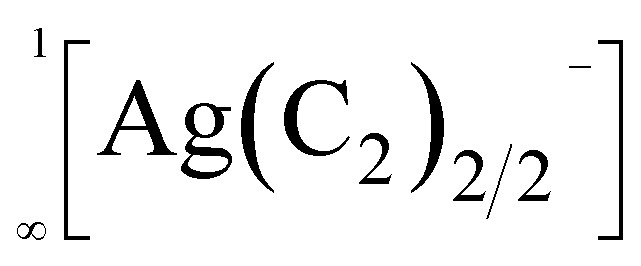
 chains, which are separated by K^+^ cations.^9^ Similar 
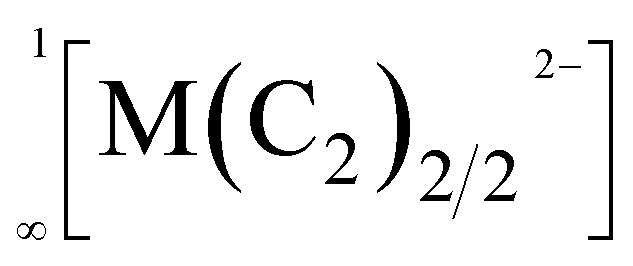
 chains were found in compounds with the composition A_2_MC_2_ (A = Na–Cs; M = Pd, Pt).^10–12^ All these chain-type structures are not unexpected, as Ag(i) as well as Pd(0) and Pt(0) feature a *d*^10^ electron configuration, for which a linear coordination, as found in these chains, is very common.^13,14^

Therefore, it was very surprising, when back in 2015 Németh *et al.* proposed similar linear chain-type structures 
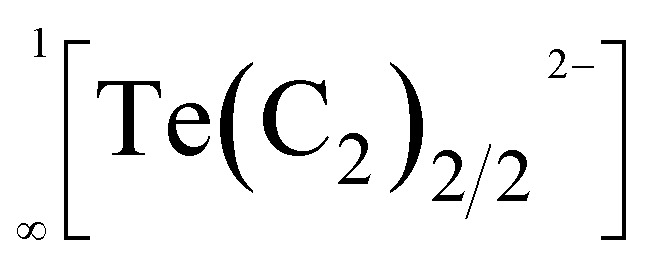
 within the acetylides Li_2_TeC_2_ and Na_2_TeC_2_.^15^ Te(0), which should exist in these compounds, is not known to prefer a linear coordination. Quite the contrary, in elemental tellurium a tilted arrangement with Te–Te–Te angles of approx. 102.3° was reported.^16^ Nonetheless, the ternary tellurium acetylides Li_2_TeC_2_ and Na_2_TeC_2_ seem to be intriguing materials with several potential applications as photocathodes or novel electrode materials for batteries, as predicted on the basis of density functional theory (DFT) calculations.^17^ They inspired us to have a closer look at acetylides with the main group elements sulphur, selenium, and tellurium. In these works, we were able to synthesise and characterise the elusive and mainly unprecedented anions ^−^S–C≡C–H,^18^^−^Se–C≡C–H,^19,20^ and ^−^Se–C≡C–Se^−^.^21^ However, we have never been able to observe a chain-type structure as published by Németh *et al.*^15^ With regard to the present work, it is important to note that the above-mentioned anions typically occur in an ordered arrangement in their crystal structures. Only in NaSC_2_H^18^ and NaSeC_2_H,^20^ the anions show an orientational disorder over two positions. Also at low temperatures down to 100 K, no ordering was observed.

One obvious drawback of the work by Németh *et al.* is the very low crystallinity of Li_2_TeC_2_ and Na_2_TeC_2_, resulting in X-ray powder diffraction patterns with only very few and very broad reflections, from which their crystal structures had to be solved and refined (Li_2_TeC_2_: P3̄*m*1; Na_2_TeC_2_: *I*4/*mmm*).^15^ In our work on selenium acetylides, we observed very similar diffraction patterns after heating ASeC_2_H (A = Na–Cs) in a dynamic vacuum. In contrast to the work of Nemeth *et al.*,^15^ we were able to index these patterns in small cubic unit cells.^19^ As the release of acetylene (C_2_H_2_) was proven during heating, we postulated a reaction according to^20^
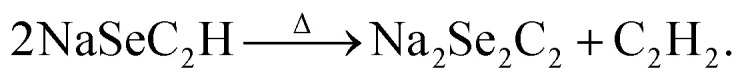


This assumption would challenge the findings of Németh *et al.* At this point, it is notable that their DFT calculations on Li_2_TeC_2_ predicted a much smaller unit cell with an inverted *c*/*a* ratio, already pointing out possible inaccuracies in their structural model.^15^ In the following, we will give a clear proof that the cubic selenium compounds we found in our investigations^19^ have the composition A_2_Se_2_C_2_ and contain ^−^Se–C≡C–Se^−^ dianions, which have been observed before in Li_2_Se_2_C_2_·2NH_3_.^21^ Due to orientational disorder of these anions on a local scale, the reflections are broadened and PDF (pair distribution function) analysis accompanied by DFT calculations is needed to develop a reasonable structural model for this disorder. In our view, this approach provides an engaging example of how reliable structural information can be obtained even from a poorly crystalline sample by combining PDF analyses and DFT calculations with conventional spectroscopic studies and Rietveld refinements.

## Experimental

### Synthesis

ASeC_2_H (A = Na–Cs) was synthesised according to a previously published method.^19,20^ The resulting compound was transferred to a Schlenk tube and heated at 200 °C for 1 h under a dynamic vacuum.

Alternatively, a mixture of A_2_C_2_ (A = Na–Cs) and two equivalents of grey selenium was suspended in liquid ammonia. The suspension was stirred for 1 h at −78 °C, after which the ammonia was evaporated at room temperature.

In both cases, the product was obtained in the form of a brown powder. The SI provides more details regarding the syntheses.

### Vibrational spectroscopy

IR spectra were measured using a Bruker ALPHA FT-IR spectrometer. Measurements were carried out inside a glovebox under inert conditions (argon atmosphere).

Raman analysis was performed using a Renishaw inVia Qontor® Raman Microscope. Samples were transferred into glass capillaries sealed under argon before the measurements. Spectra were measured using a laser wavelength of 457 nm and a Centrus 05 TJ CCD detector.

All vibrational spectra were measured on samples synthesised in liquid ammonia.

### SEM/EDX

The morphology and elemental distribution were analyzed using a Zeiss Sigma 300 VP Rise field emission SEM with an integrated Oxford Instruments Xplore 30 EDX. SEM data was measured at 2 kV, EDX data was measured at 15 kV. Samples were transferred onto a carbon grid under inert conditions (argon atmosphere) prior to the measurement.

### Powder diffraction

High-resolution synchrotron powder patterns were recorded at the DELTA facility (Dortmund, Germany), beamline BL9.^22^ Samples were measured at ambient conditions (25 keV, Dectris PILATUS 100K detector, glass capillaries *⌀* = 0.7 mm sealed under argon). All samples were synthesised in liquid ammonia.

Total scattering data was collected at the German Electron Synchrotron (DESY, Hamburg (Germany), beamline P21.1).^23^ Samples were measured under ambient conditions (101.35(10) keV, PerkinElmer XRD1621 detector, glass capillaries *⌀* = 1.0 mm sealed under argon). The 2D data was integrated using pyFAI,^24^ pair distribution functions were calculated using PDFgetX3 ^25^ on a Nyquist-Shannon grid^26^ with *Q*_max_ = 22.9 Å^−1^. Na_2_Se_2_C_2_ and K_2_Se_2_C_2_ were synthesised in liquid ammonia, while Rb_2_Se_2_C_2_ and Cs_2_Se_2_C_2_ were synthesised by heating RbSeC_2_H and CsSeC_2_H, respectively. This ensured phase purity for all samples.

Simultaneous Rietveld and pair distribution function refinements were performed using TOPAS-Academic Version 6.^27–29^ To obtain a smooth convergence, the C≡C distance was soft restrained to 1.20(2) Å and the Se–C distance to 1.85(2) Å, as observed in KSeCN.^30^ Alkali metal cations were fixed to a primitive cubic lattice and Se and C atoms were restrained so that they could only move along the Se⋯Se axis in an anion.

### Quantum chemical calculations

Solid state DFT calculations were performed using Quantum ESPRESSO.^31,32^ For total energy calculations and geometry optimizations, the PBEsol functional,^33^ pseudopotentials from the SSSP PBEsol Precision v1.3.0 library^34–38^ and D3 corrections^39^ were employed. For calculations of spectroscopic data, the LDA functional^40^ was used in combination with ONCVPSP v0.4.1 pseudopotentials^41^ from PseudoDojo.^37^

Gas phase DFT calculations were performed using ORCA,^42–47^ the B3LYP hybrid functional,^48–51^ ma-def2-QZVPP^52,53^ basis sets and D4 corrections.^54–56^ Bonds within the calculated molecules were analysed using JANPA.^57,58^ Further details regarding these calculations are available in the SI.

## Results and discussion

### Structural characterisation

**Fig. 1:**
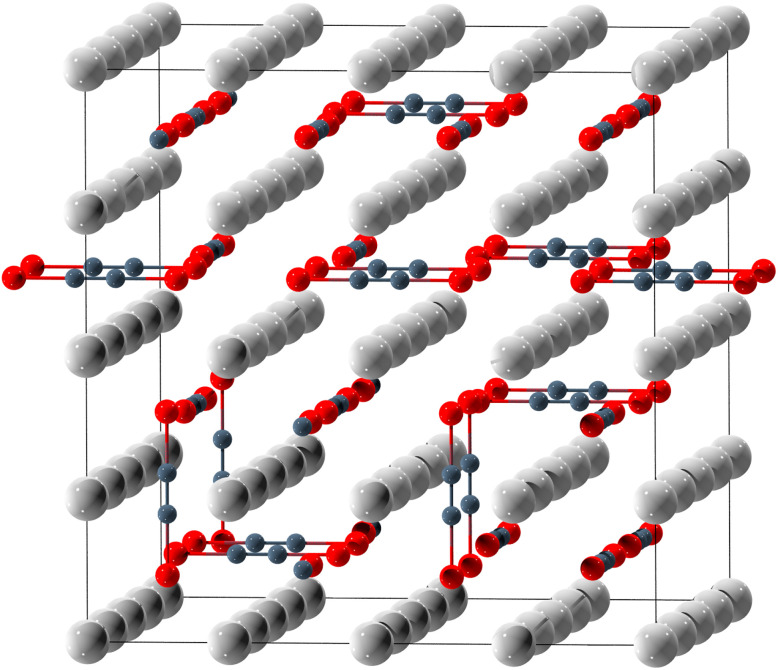
4 × 4 × 4 supercell of K_2_Se_2_C_2_ with disordered ^−^Se–C≡C–Se^−^ anions. Colour code: K: light grey, C: dark grey, Se: red.

Although the PXRD patterns of A_2_Se_2_C_2_ (A = Na–Cs) can be indexed in small, primitive cubic unit cells,^19,20^ a complete ordering of the linear ^−^Se–C≡C–Se^−^ anions within these small unit cells is not possible. Similar behaviour has already been observed for CsSeH, which appears to crystallise in the CsCl structure type with obviously rotationally disordered SeH^−^ anions, according to the analysis of Bragg diffraction data.^59^ For this reason, a structural model of orientationally disordered anions in the *a*, *b*, and *c* directions was envisaged for the compounds A_2_Se_2_C_2_. For the Rietveld and PDF fits, a 4 × 4 × 4 supercell of the primitive cubic unit cell containing statistically disordered anions (Fig. 1) was constructed for each compound. Statistical, orientational disorder was simulated using the Metropolis–Hastings algorithm^60^ with local edge flips in a Markov chain Monte Carlo simulation^61^ comprising 10 000 steps.

**Fig. 2:**
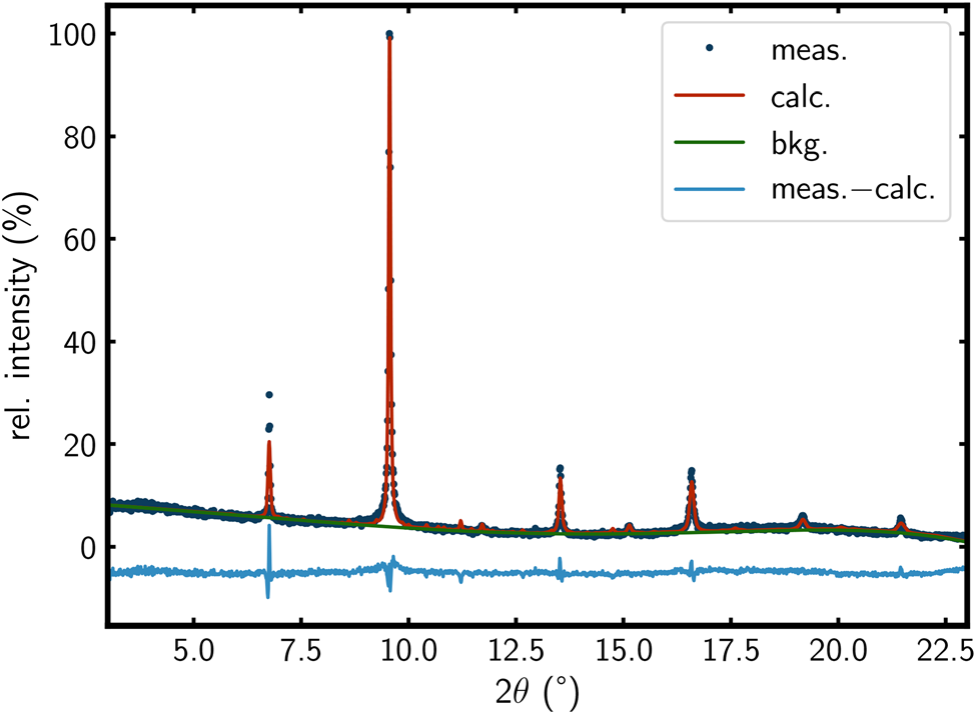
Rietveld fit of K_2_Se_2_C_2_ in a 4 × 4 × 4 supercell (Fig. 1) with disordered anions (*λ* = 0.496 Å).

The Rietveld fits of all A_2_Se_2_C_2_ compounds are in good agreement with the PXRD measurements (Fig. 2; S1, S3, S5, SI). The Rietveld fit of Rb_2_Se_2_C_2_ shows small amounts of RbSeC_2_H^19^ impurities. This suggests an analogous equilibrium for the anion couple SeC_2_H^−^/Se_2_C_2_^2−^ as found for C_2_H^−^/C_2_^2−^ in liquid ammonia.^62^

**Fig. 3:**
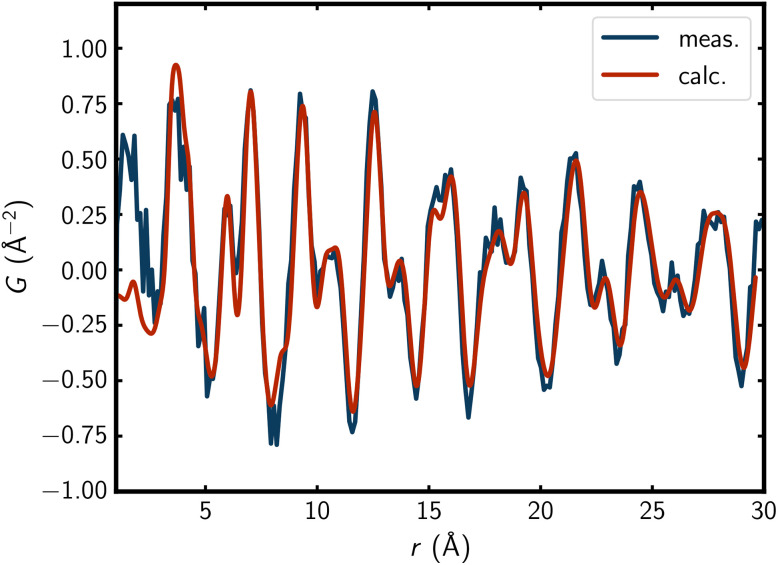
PDF fit of K_2_Se_2_C_2_ in a 4 × 4 × 4 supercell (Fig. 1) with disordered anions (*Q*_max_ = 22.9 Å^−1^).

The PDF fits of the A_2_Se_2_C_2_ compounds also show a good agreement with the experimental PDFs (Fig. 3; Fig. S2, S4, S6, SI). The only major discrepancies appear in the very low *r*-region, where the experimental PDFs are significantly higher than the fits. This is most likely a result of termination ripples^63^ caused by the cutoff at *Q*_max_ = 22.9 Å^−1^ as well as some noise in *F*(*Q*) at high *Q*, and is therefore not indicative of any structural features.

**Fig. 4:**
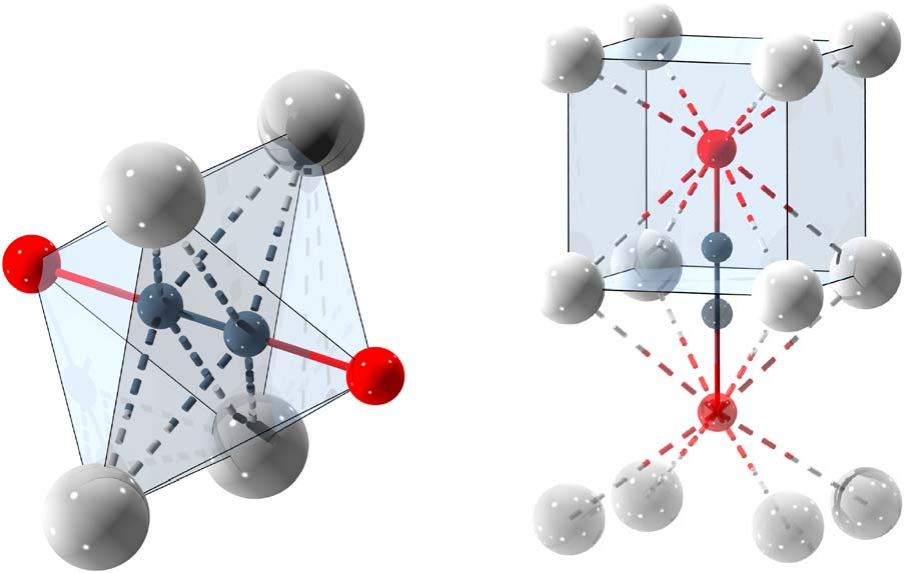
Coordination sphere of the ^−^Se–C≡C–Se^−^ anion in K_2_Se_2_C_2_. Left: side-on coordination of the C≡C unit. Right: Se sitting in the cubic K_8_ void. Colour code: K: light grey, C: dark grey, Se: red.

Each Se_2_C_2_^2−^ anion bridges two cubes spanned by the alkali metal cations (Fig. 4 right). The acetylidic C≡C dumbbell is orientated perpendicular to the square face that connects the two cubes and is coordinated by four cations in a side-on fashion (Fig. 4 left).

The Se atoms sit within the cubic voids spanned by the alkali metal cations. They are slightly shifted out of the cube's centre along the Se–C axis (Fig. 4 right). The distortion from the coordination within a platonic cube is small and decreases with a larger cation radius and, consequently, larger unit cell parameters (Table S2, SI), as would be expected. The intraanionic Se⋯Se distance is 4.90(3) Å and the alkali metal distance (*i.e.* the lattice parameter of the primitive unit cell) approaches this value for the larger alkali metals. The alkali metal-selenium distances are consistently longer than those found in reference compounds (Table 1), indicating weaker interactions.

**Table 1:** Selected interatomic distances in A_2_Se_2_C_2_ and comparison with reference compounds A_2_Se

	*d*_A–Se_ (short)	*d*_A–Se_ (long)	*d*_A–Se_ (A_2_Se)
Na_2_Se_2_C_2_	3.1627(14) Å, 4×	3.6884(12) Å, 4×	2.96 Å, 8× ^64^
K_2_Se_2_C_2_	3.4989(5) Å, 4×	3.8469(5) Å, 4×	3.33 Å, 8× ^64^
Rb_2_Se_2_C_2_	3.618(5) Å, 4×	3.924(5) Å, 4×	3.47 Å, 8× ^65^
Cs_2_Se_2_C_2_	3.85(7) Å, 4×	4.03(8) Å, 4×	3.51 Å, 8× ^65^

To assess the hypothesis of disordered ^−^Se–C≡C–Se^−^ anions, a structural model with ordered anions was also fitted to the data. This model belongs to the tetragonal space group type *P*4/*mmm* (no. 123) and contains two crystallographically independent alkali metal cations, with the anion aligned along the *c* axis (Fig. S7, SI). However, with the exception of the Rb compound, this model consistently provides a poorer fit to the data (Table 2). The difference in *R*_wp_ values for Rb_2_Se_2_C_2_ is negligible and does not prefer any of the possible models significantly. However, a model of locally disordered anions also in Rb_2_Se_2_C_2_ is supported by DFT calculations and Raman spectroscopy, as will be demonstrated in the following.

**Table 2:** *R*_wp_ values for combined Rietveld and PDF fits of A_2_Se_2_C_2_ for two different models

	*R*_wp_ 4 × 4 × 4 (%)	*R*_wp_ *P*4/*mmm* (%)
Na_2_Se_2_C_2_	12.35	16.06
K_2_Se_2_C_2_	8.46	12.13
Rb_2_Se_2_C_2_	6.50	5.84
Cs_2_Se_2_C_2_	6.94	8.65

The tetragonal structure model with ordered anions shows clear deviations in reflection intensities as well as additional reflections in the Rietveld fit. For example, there are additional reflections for K_2_Se_2_C_2_ between 10° and 13° (Fig. S9, SI). These errors are significantly smaller in the Rietveld fit of the 4 × 4 × 4 supercell containing disordered anions (Fig. S8, SI). The remaining discrepancies are probably due to the finite size of the supercell. However, refinement of even larger supercells was not computationally feasible.

**Fig. 5:**
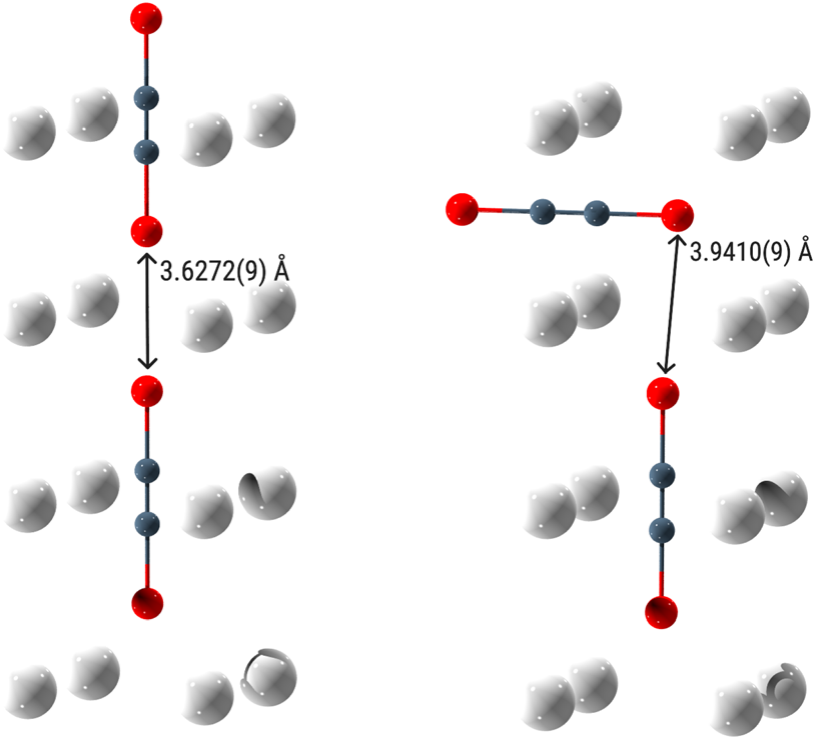
Interanionic distance between Se atoms in K_2_Se_2_C_2_. Left: ordered anions (*P*4/*mmm*). Right: disordered anions (4 × 4 × 4 supercell). Colour code: K: light grey, C: dark grey, Se: red.

This evident orientational disorder of the anions may be rationalised by the resulting increase of the intermolecular anion–anion distances. If the anions were ordered, the partially negatively charged Se atoms would approach each other along the *c* axis (Fig. 5 left). For K_2_Se_2_C_2_*e.g.*, this would result in a Se⋯Se distance of 3.6272(9) Å. Notably, this is less than twice the van der Waals radius of selenium, which is 1.9 Å.^66^ If the anions are orientationally disordered, only some anions directly point at each other like in the model with *P*4/*mmm* symmetry (Fig. 5 left), while most of them evade each other with respect to short Se⋯Se distances (Fig. 5 right). For the K_2_Se_2_C_2_ supercell (Fig. 1), this results in an average Se⋯Se distance of 3.810(4) Å with a mode (*i.e.* the most common value) of 3.9410(9) Å. This now is more than twice the van der Waals radius of selenium.

### SEM/EDX

**Fig. 6:**
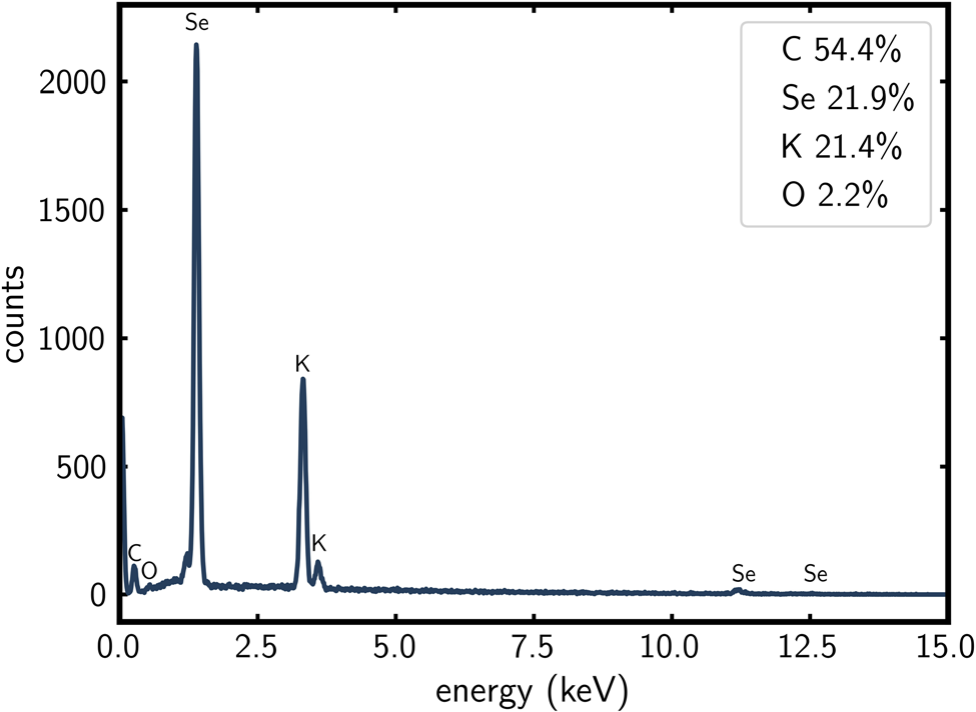
EDX spectrum of K_2_Se_2_C_2_ with atomic percentages for C, Se, K and O.

The EDX (energy-dispersive X-ray spectroscopy) spectrum of K_2_Se_2_C_2_ demonstrates a K : Se ratio of 1 : 1 within experimental errors (Fig. 6), which further indicates that its composition is A_2_Se_2_C_2_. The C signals are stronger than expected as the measurement was carried out on a carbon grid. The presence of small amounts of oxygen in the sample is probably due to a slight oxidation during transfer into the device. SEM (scanning electron microscopy) images of the sample demonstrate the cubic morphology of K_2_Se_2_C_2_ (Fig. S10, SI).

### Solid state DFT calculations

**Fig. 7:**
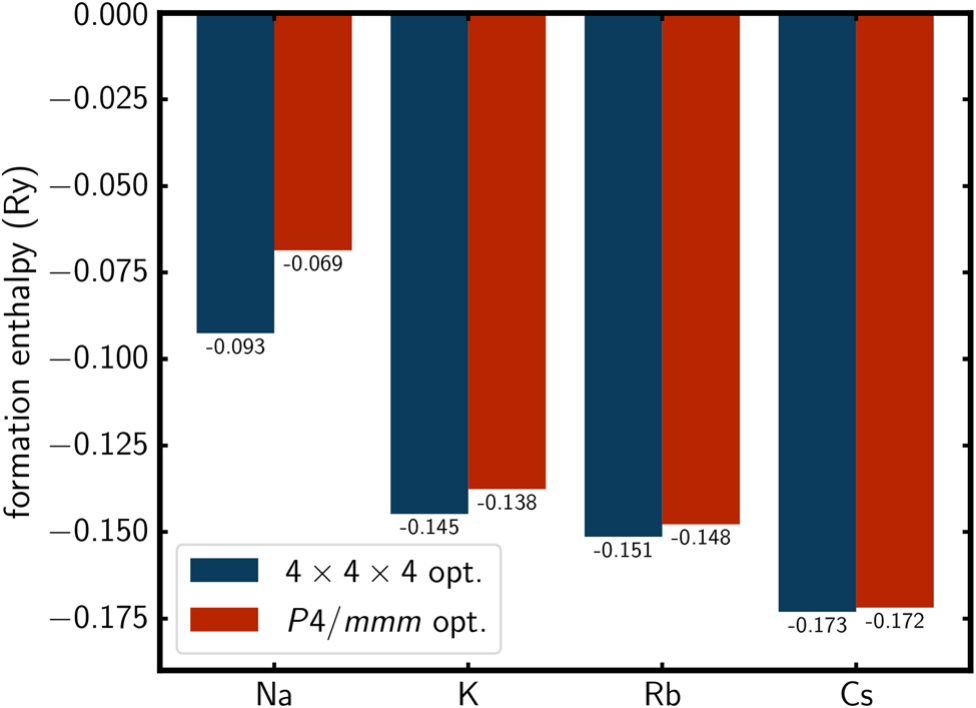
Formation enthalpies per atom for A_2_Se_2_C_2_ compounds after optimisation in a 4 × 4 × 4 supercell and in *P*4/*mmm* symmetry.

To further analyse the structural motif of the A_2_Se_2_C_2_ compounds, the disordered model in a 4 × 4 × 4 supercell and the ordered model with *P*4/*mmm* symmetry were optimised using DFT. The resulting formation enthalpies show that all structures are thermodynamically stable compared to the elements and that the disorder of the anions leads to a favourable energy gain (Fig. 7). This effect decreases from Na to Cs, which further hints at the increase of Se⋯Se distances as the driving force for the orientational disorder. As the alkali metal radii and subsequently the lattice parameters increase, the Se⋯Se distances increase as well. As a result, even if the anions point at each other in the ordered *P*4/*mmm* model, the interanionic Se⋯Se distances are larger than twice the van der Waals radius of selenium for the larger alkali metal cations.

Furthermore, the lattice parameters of the orientationally disordered model of A_2_Se_2_C_2_ barely decreased during optimisation (Table S3–S6, SI), which is common for these calculations.^67^ The unit cell angles all stayed very close to 90°. In contrast, the *c* parameter of the *P*4/*mmm* model changed significantly during optimisation, leading to large distortions of the pseudo-cubic cell (Table S3–S6, SI).

### Vibrational spectroscopy

The IR spectra clearly show modes from 740 cm^−1^ to 768 cm^−1^ for all A_2_Se_2_C_2_ (A = Na–Cs) compounds (Fig. S11, SI). These can be attributed to the Se–C stretching vibration, which can be reproduced using solid-state density functional perturbation theory (DFPT)^68^ (Table 3). The absence of modes characteristic of ammonia clearly shows that, in contrast to lithium,^21^ no ammoniate is formed for A = Na–Cs. As already observed in the PXRD data, weak modes that can be attributed to minor impurities of RbSeC_2_H and CsSeC_2_H, respectively, are evident in the IR spectra of Rb_2_Se_2_C_2_ and Cs_2_Se_2_C_2_.

**Table 3:** Experimental and calculated IR and Raman shifts of significant stretching vibrations in A_2_Se_2_C_2_ (A = Na–Cs). IR and Raman shifts of Li_2_Se_2_C_2_·2NH_3_ ^21^ are given for reference

	*ν*_Se–C,exp _	*ν*_Se–C,calc_	*ν*_C≡C,exp _	*ν*_C≡C,calc_
Na_2_Se_2_C_2_	754 cm^−1^	754.24 cm^−1^	1977 cm^−1^	1970.88 cm^−1^
K_2_Se_2_C_2_	768 cm^−1^	765.32 cm^−1^	2004 cm^−1^	1998.04 cm^−1^
Rb_2_Se_2_C_2_	767 cm^−1^	761.17 cm^−1^	2009 cm^−1^	2004.32 cm^−1^
Cs_2_Se_2_C_2_	740 cm^−1^	748.95 cm^−1^	2014 cm^−1^	2007.10 cm^−1^
Li_2_Se_2_C_2_·2NH_3_	756 cm^−1^	760 cm^−1^	2059 cm^−1^	2057 cm^−1^

**Fig. 8:**
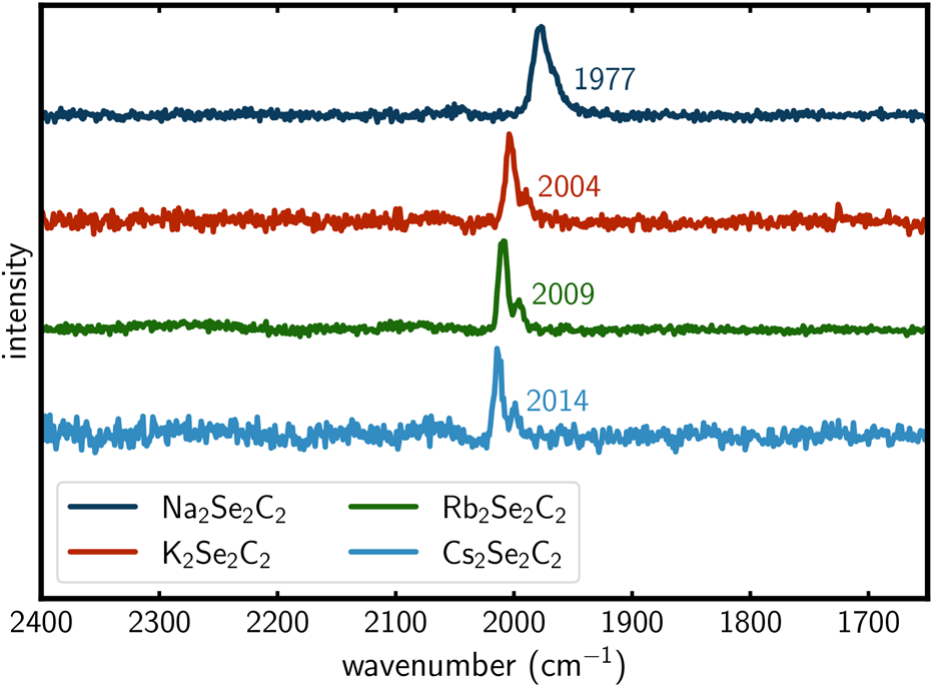
Raman spectra of the A_2_Se_2_C_2_ (A = Na–Cs) compounds. Wavenumbers of the C≡C stretching vibration are given.

The Raman spectra show modes that can be assigned to the C≡C stretching vibration. The modes for K_2_Se_2_C_2_, Rb_2_Se_2_C_2_ and Cs_2_Se_2_C_2_ are all very similar, with wavenumbers ranging from 2004 cm^−1^ to 2014 cm^−1^, whereas the mode for Na_2_Se_2_C_2_ is shifted to 1977 cm^−1^ (Fig. 8). These results could be reproduced in solid-state DFPT^68^ calculations (Table 3). All Raman spectra show a shoulder (A = Na) or a second signal at lower wavenumbers (A = K, Rb, Cs). These are caused by the two different possible interanionic arrangements (Fig. 5). As the disordered structure model contains both motifs (*vide supra*), the C≡C stretching vibration leads either to the anions swinging directly into each other (Fig. 5 left) or swinging side by side (Fig. 5 right), resulting in slightly different Raman modes. Both the IR and the Raman modes of the ^−^Se–C≡C–Se^−^ anion are consistent with the modes found for Li_2_Se_2_C_2_·2NH_3_ (Table 3).^21^

Gas phase DFT calculations reveal a Raman mode of 2080.97 cm^−1^ for the formal ^−^Se–C≡C–Se^−^ dianion and a mode of 2013.93 cm^−1^ for the formal ^.^Se–C≡C–Se^−^ radical monoanion. This difference might explain the shifted Raman mode for Na_2_Se_2_C_2_. As Na (5.139 eV) has a significantly higher first ionization energy than K (4.341 eV), Rb (4.177 eV) and Cs (3.894 eV),^69^ the electronic structure of the anion in Na_2_Se_2_C_2_ will be closer to a formal monoanion, whereas the similar first ionization energies of K, Rb and Cs lead to comparable Raman shifts for these compounds. This is most likely caused by a weaker C≡C bond in the slightly electron deficient monoanion compared to the dianion. This weaker bond in Se_2_C_2_^−^ compared to Se_2_C_2_^2−^ is corroborated by CLPO^58^ bond analysis. This analysis indicates a bond order of 2.78 for the dianion, but only 2.58 for the monoanion, as the additional electron of the dianion mainly occupies the bonding π-orbital of the C_2_ unit.

## Conclusions

We were able to synthesise polycrystalline A_2_Se_2_C_2_ (A = Na–Cs) by either reacting A_2_C_2_ with grey selenium in liquid ammonia, or by heating ASeC_2_H. Their crystal structures were modelled and solved from synchrotron powder data and pair distribution functions. The resulting structure features a primitive cubic lattice of alkali metal cations with Se_2_C_2_^2−^ anions positioned between them. The presence of the anions is corroborated by IR and Raman spectra. The anions are orientationally disordered along the crystallographic *a*, *b*, and *c* axes. Models with ordered Se_2_C_2_^2−^ anions resulted in poorer fits of PXRD and PDF data, and reduced stability according to DFT calculations.

As a continuation of this work, we plan to investigate the corresponding tellurium-containing compounds. As previously demonstrated,^19^ we were able to synthesise these compounds from A_2_C_2_ (A = Na, K, Rb) and elemental tellurium, which show similar PXRD patterns as those presented in this work. The resulting patterns could be indexed in small primitive cubic unit cells, *e.g. a* = 4.144 Å for the sodium compound.^19^ It is in our opinion very likely, that these Te compounds exhibit similar structures as presented for the Se compounds in this work, which would challenge the results of Németh *et al.*^15^ Unfortunately, the crystal structures of these compounds could not be fully elucidated until now, partly due to their increased chemical and mechanical sensitivity.

## Author contributions

T. M.: data curation, investigation, methodology, software, visualisation, writing – original draft. M. H.: conceptualisation, data curation, investigation, methodology, writing – review & editing. U. R.: funding acquisition, resources, supervision, writing – review & editing.

## Conflicts of interest

There are no conflicts to declare.

## Supplementary Material

RA-016-D6RA01467D-s001

RA-016-D6RA01467D-s002

## Data Availability

Data supporting this article, has been included as part of the supplementary information (SI). Supplementary information: Tables S1–S7, further Rietveld and PDF fits, IR spectra, further experimental and computational details. IR and Raman data for this article are available at Chemotion Repository at https://dx.doi.org/10.14272/collection/tmattic2_2026-02-04. The code for the supercell generation is available at https://doi.org/10.5281/zenodo.18611408. See DOI: https://doi.org/10.1039/d6ra01467d. CCDC 2529945–2529948 contain the supplementary crystallographic data for this paper.^70*a*–*d*^
