# Supplementary material for: A2Se2C2 (A = Na–Cs): crystalline compounds featuring −Se–C[triple bond, length as m-dash]C–Se− dianions with short-range structural disorder
Source: RSC Adv. 2026 May 5;16(25):23037–43. doi: 10.1039/d6ra01467d (PMC13142230; doi:10.1039/d6ra01467d)
Supplement: RA-016-D6RA01467D-s001 [file RA-016-D6RA01467D-s001.pdf]

Supplementary Information (SI) for RSC Advances.
This journal is © The Royal Society of Chemistry 2026

Supplementary Information

$A_2Se_2C_2$ (A = Na–Cs): Crystalline Compounds Featuring $^{-}Se{-}C{\equiv}C{-}Se^{-}$ Dianions with Short-Range Structural Disorder

T. Mattick, M. Hetzert, U. Ruschewitz

Contents

1 Synthesis of $A_2Se_2C_2$ S2
1.1 Synthesis in liquid ammonia . . . S2
1.2 Synthesis by heating of $ASeC_2H$ . . . S2

2 Structural characterisation S2
2.1 Pair Distribution Functions . . . S2
2.2 Additional Information . . . S3
2.3 $Na_2Se_2C_2$ . . . S3
2.4 $K_2Se_2C_2$ . . . S4
2.5 $Rb_2Se_2C_2$ . . . S5
2.6 $Cs_2Se_2C_2$ . . . S6
2.7 Comparison with $P4/mmm$ cell . . . S8

3 SEM S10

4 DFT calculations S10
4.1 Solid State DFT calculations . . . S10
4.2 Gas phase DFT calculations . . . S11

5 Vibrational spectroscopy S12

References S12

1 Synthesis of $A_2Se_2C_2$

Warning These compounds are highly sensitive to impact. They may react explosively with moisture, releasing acetylene. Therefore, we strongly advise handling only small quantities of these compounds at a time.

1.1 Synthesis in liquid ammonia

$$A_2C_2 + 2Se \xrightarrow{NH_3} A_2Se_2C_2$$

A_2C_2 and two equivalents of grey Se (quantities in Tab. S1) were transferred into a Schlenk tube under argon atmosphere (glovebox). Approx. 30 ml dry NH_3 was condensed into the Schlenk tube on the vacuum line. For all alkali metals, this resulted in orange solutions that were stirred in a dry ice/isopropanol bath at −78 °C for 1 h. During this time, the selenium dissolved completely. After the reaction period, NH_3 was evaporated at room temperature and afterwards evacuated under dynamic vacuum for 30 min. The product was obtained in the form of a brown, polycrystalline powder.

Table S1 Reaction quantities for the synthesis of $A_2Se_2C_2$ in liquid ammonia.

	A_2C_2			Se		
	m (mg)	n (mmol)	Eq.	m (mg)	n (mmol)	Eq.
$Na_2Se_2C_2$	100.7	1.439	1.0	227.1	2.876	2.0
$K_2Se_2C_2$	99.8	0.976	1.0	154.5	1.956	2.0
$Rb_2Se_2C_2$	100.1	0.513	1.0	81.5	1.032	2.0
$Cs_2Se_2C_2$	100.3	0.346	1.0	55.0	0.700	2.0

1.2 Synthesis by heating of $ASeC_2H$

$$2ASeC_2H \xrightarrow{\Delta} A_2Se_2C_2 + C_2H_2$$

An unspecified amount of $ASeC_2H$ was transferred into a small glass vial under argon atmosphere. The glass vial was transferred into a Schlenk tube. The Schlenk tube was heated at a rate of $3\,°C\,min^{-1}$ under dynamic vacuum to 200 °C. The temperature was held for 1 h. The product was obtained as a brown, polycrystalline powder.

2 Structural characterisation

2.1 Pair Distribution Functions

All equations regarding the PDF or structure functions are given in the PDFFIT formalism.[1] This means that

$$G(r) = 4\pi r[\rho(r) - \rho_0] \tag{1}$$

$$= \frac{2}{\pi}\int_0^\infty F(Q)\sin(Qr)\,dQ \tag{2}$$

$$= \frac{2}{\pi}\int_0^\infty Q[S(Q)-1]\sin(Qr)\,dQ \tag{3}$$

with $\rho(r)$ being the microscopic pair density, ρ_0 the average number density, Q the magnitude of the scattering vector and $F(Q)$ the reduced structure function.[2,3] $S(Q)$ is the structure function, defined as

$$S(Q) = \frac{I_c(Q) - \langle f(Q)^2 \rangle + \langle f(Q) \rangle^2}{\langle f(Q) \rangle^2}. \tag{4}$$

$I_c(Q)$ denotes the measured coherent scattering intensity, $f(Q)$ is the atomic scattering factor. The angle brackets indicate taking the average over all atoms in the sample.[3]

2.2 Additional Information

Table S2 Deviation δ_{cube} from a perfect cubic SeA_8 (A = Na–Cs) coordination, calculated using Polynator[4].

	$Na_2Se_2C_2$	$K_2Se_2C_2$	$Rb_2Se_2C_2$	$Cs_2Se_2C_2$
δ_{cube}	4.479	2.747	2.355	1.386

2.3 $Na_2Se_2C_2$

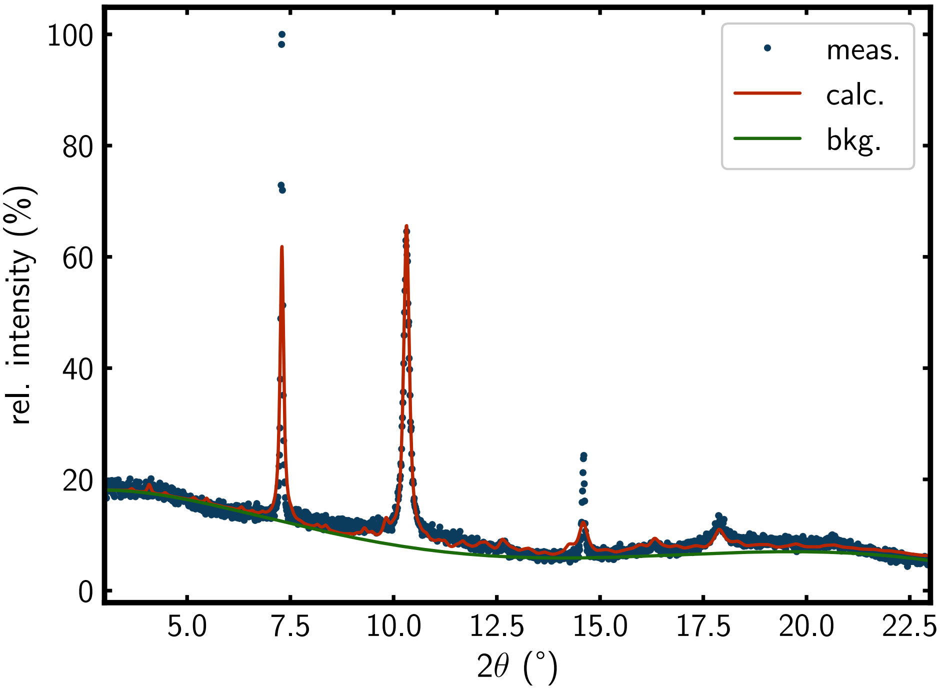

Figure S1 Rietveld fit of $Na_2Se_2C_2$ in a $4 \times 4 \times 4$ supercell with disordered anions ($\lambda = 0.496\,\text{Å}$).

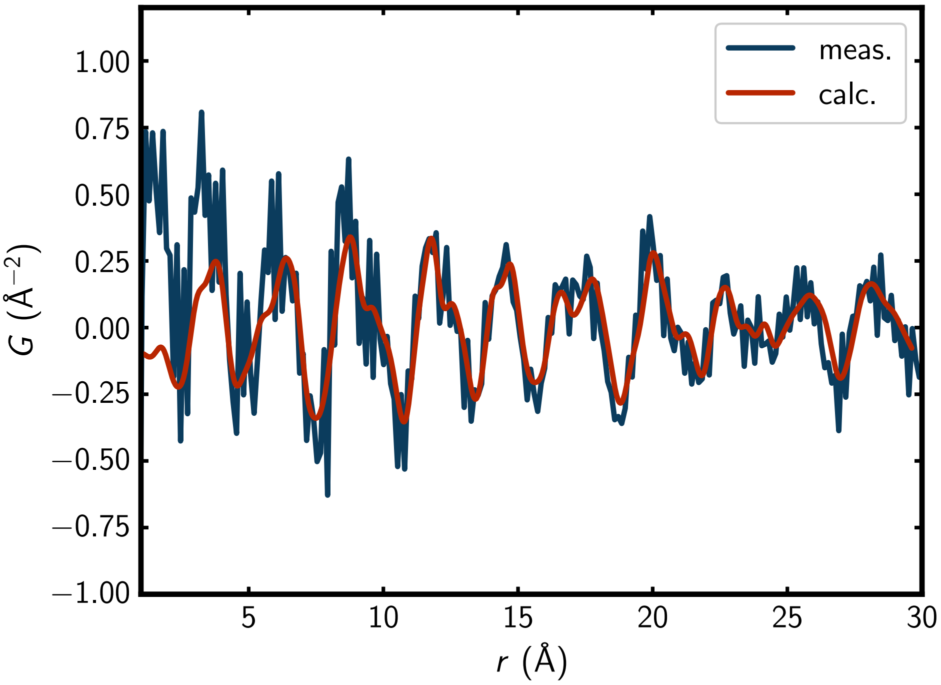

Figure S2 PDF fit of $Na_2Se_2C_2$ in a $4 \times 4 \times 4$ supercell with disordered anions ($Q_{\mathrm{max}} = 22.9\,\mathrm{\AA}^{-1}$).

Table S3 Lattice parameters of $Na_2Se_2C_2$ obtained from combined Rietveld and PDF fits as well as from DFT calculations.

	$4 \times 4 \times 4$		$P4/mmm$	
	refined	calculated	refined	calculated
a (Å)	15.727(9)	15.536	3.92(3)	3.903
b (Å)	15.727(9)	15.454	3.92(3)	3.903
c (Å)	15.727(9)	15.507	7.71(7)	7.938
$\frac{c}{a}$	1	0.998	1.967(23)	2.034
α (°)	90	90.014	90	90
β (°)	90	89.578	90	90
γ (°)	90	91.059	90	90

2.4 $K_2Se_2C_2$

Table S4 Lattice parameters of $K_2Se_2C_2$ obtained from combined Rietveld and PDF fits as well as from DFT calculations.

	$4 \times 4 \times 4$		$P4/mmm$	
	refined	calculated	refined	calculated
a (Å)	16.926(4)	16.461	4.223(3)	4.246
b (Å)	16.926(4)	16.929	4.223(3)	4.246
c (Å)	16.926(4)	16.556	8.449(7)	8.150
$\frac{c}{a}$	1	1.006	2.001(2)	1.919
α (°)	90	89.675	90	90
β (°)	90	89.936	90	90
γ (°)	90	89.979	90	90

2.5 $Rb_2Se_2C_2$

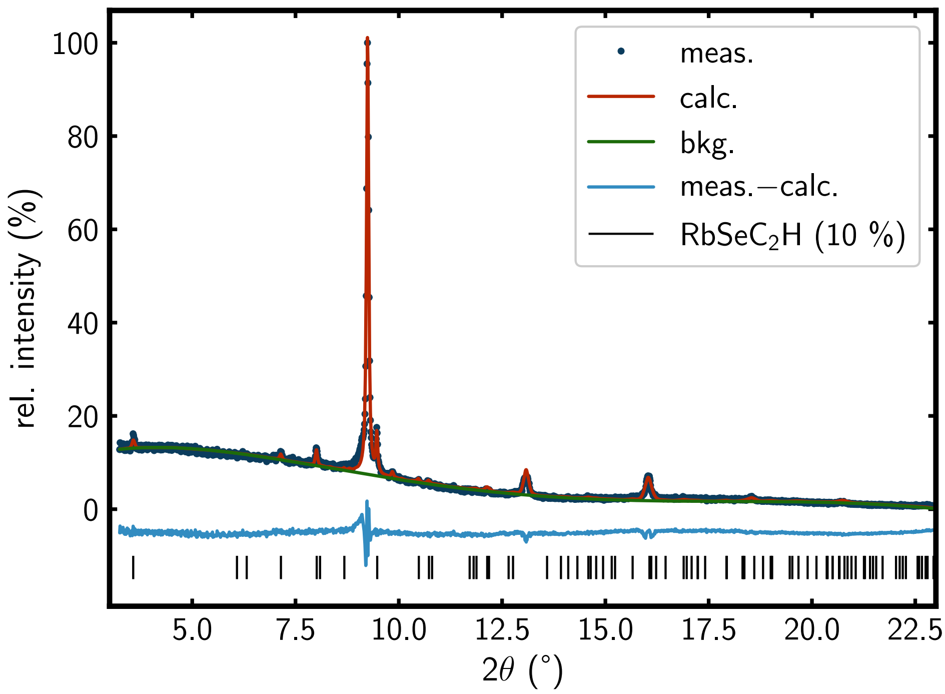

Figure S3 Rietveld fit of $Rb_2Se_2C_2$ in a $4 \times 4 \times 4$ supercell with disordered anions ($\lambda = 0.496\,\text{Å}$). The amount of the impurity $RbSeC_2H$[5] was fitted to $10\,\%$, marked with vertical bars.

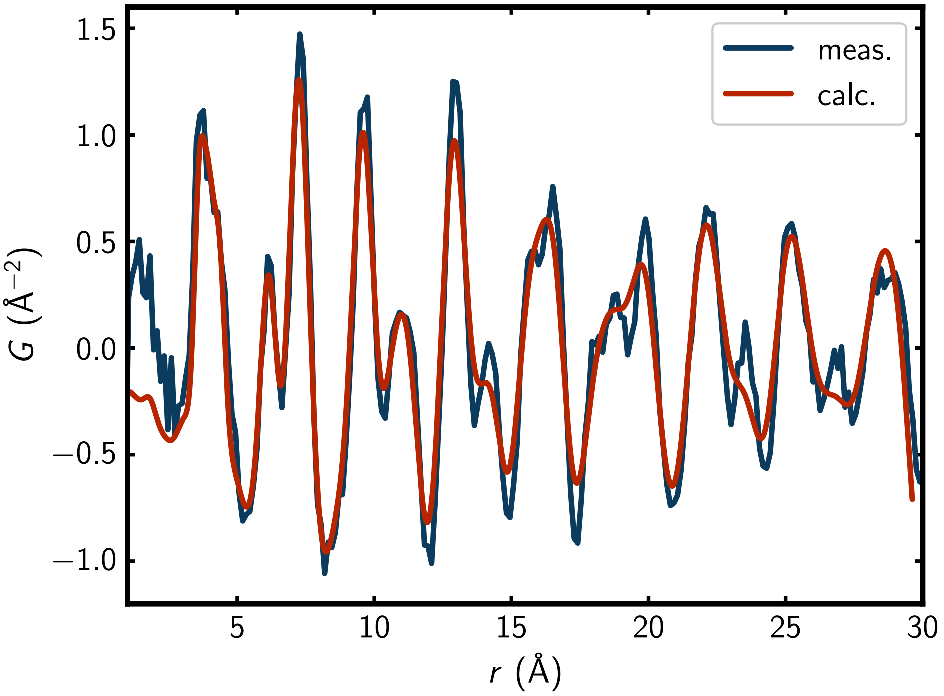

Figure S4 PDF fit of $Rb_2Se_2C_2$ in a $4 \times 4 \times 4$ supercell with disordered anions ($Q_{\text{max}} = 22.9\,\text{Å}^{-1}$).

Table S5 Lattice parameters of $Rb_2Se_2C_2$ obtained from combined Rietveld and PDF fits as well as from DFT calculations.

	$4 \times 4 \times 4$		$P4/mmm$	
	refined	calculated	refined	calculated
a (Å)	17.391(4)	17.136	4.378(8)	4.421
b (Å)	17.391(4)	17.226	4.378(8)	4.421
c (Å)	17.391(4)	17.311	8.805(17)	8.261
$\frac{c}{a}$	1	1.010	2.011(5)	1.869
α (°)	90	90.040	90	90
β (°)	90	90.222	90	90
γ (°)	90	90.054	90	90

2.6 $Cs_2Se_2C_2$

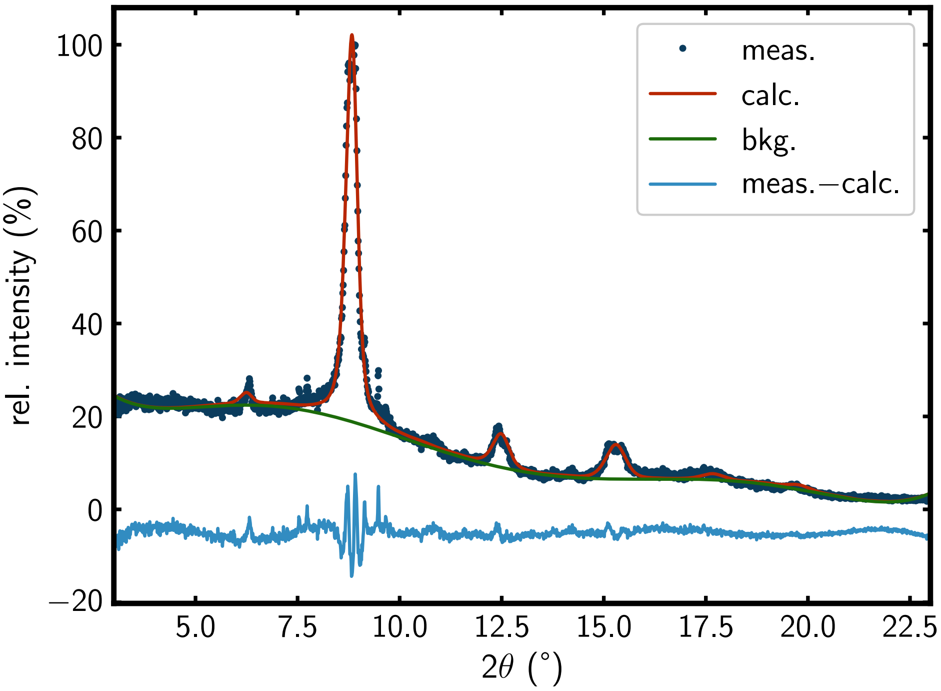

Figure S5 Rietveld fit of $Cs_2Se_2C_2$ in a $4 \times 4 \times 4$ supercell with disordered anions ($\lambda = 0.496\,\text{Å}$).

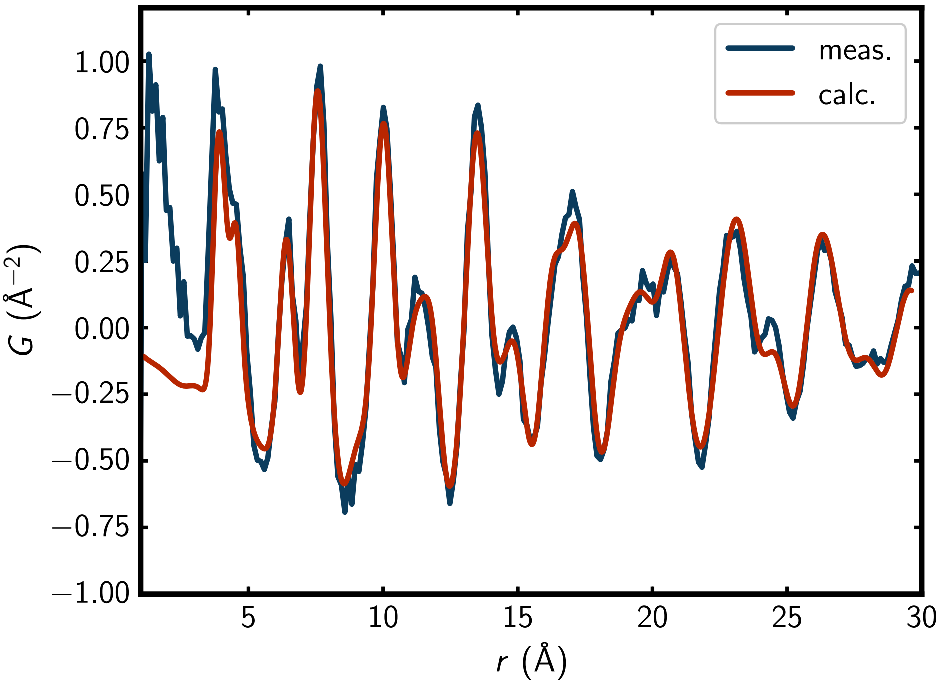

Figure S6 PDF fit of $Cs_2Se_2C_2$ in a $4 \times 4 \times 4$ supercell with disordered anions ($Q_{\max} = 22.9\,\text{Å}^{-1}$).

Table S6 Lattice parameters of $Cs_2Se_2C_2$ obtained from combined Rietveld and PDF fits as well as from DFT calculations.

	$4 \times 4 \times 4$		$P4/mmm$	
	refined	calculated	refined	calculated
a (Å)	18.211(12)	18.136	4.599(16)	4.622
b (Å)	18.211(12)	17.789	4.599(16)	4.622
c (Å)	18.211(12)	17.807	8.92(3)	8.397
$\frac{c}{a}$	1	0.982	1.940(9)	1.817
α (°)	90	89.926	90	90
β (°)	90	89.873	90	90
γ (°)	90	89.895	90	90

2.7 Comparison with $P4/mmm$ cell

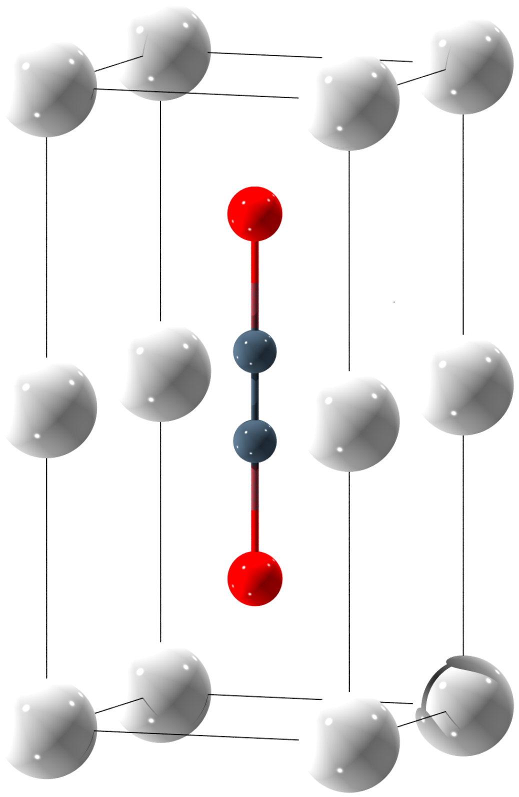

Figure S7 Potential unit cell of $K_2Se_2C_2$ with anions oriented along the c axis, crystallising in space group type $P4/mmm$.

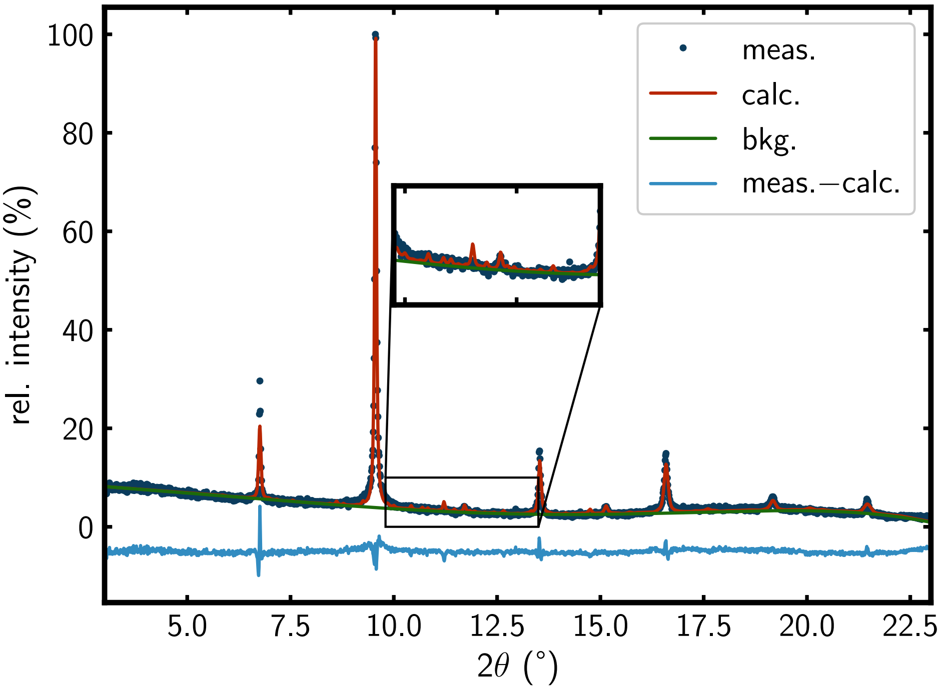

Figure S8 Rietveld fit of $K_2Se_2C_2$ in a $4 \times 4 \times 4$ supercell with disordered anions ($\lambda = 0.496\,\text{Å}$) with an inset from $9.8°$ to $13.5°$.

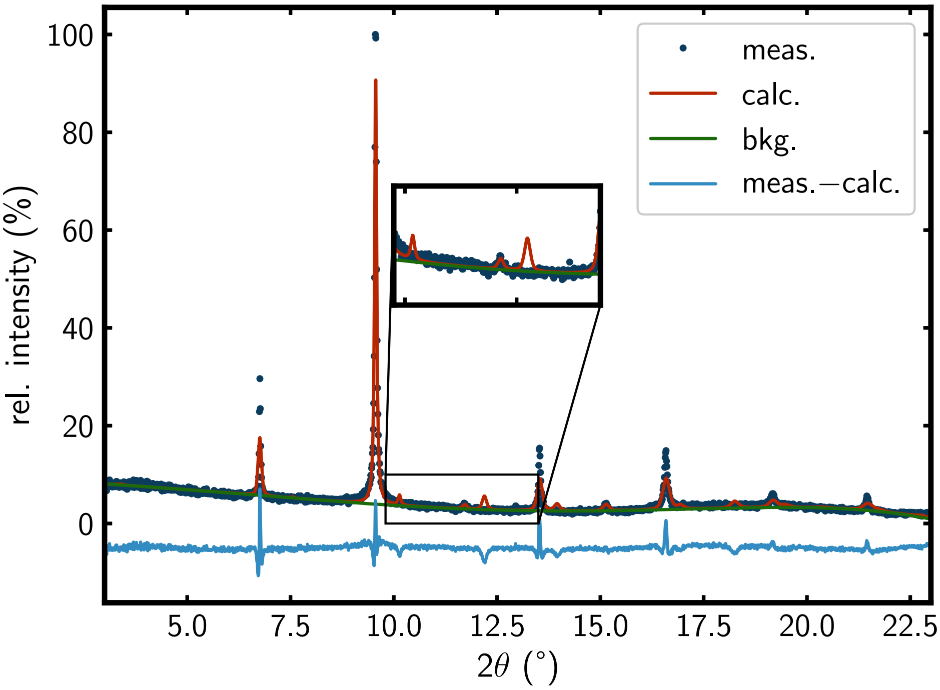

Figure S9 Rietveld fit of $K_2Se_2C_2$ in space group $P4/mmm$ with ordered anions ($\lambda = 0.496\,\text{Å}$) with an inset from $9.8°$ to $13.5°$.

3 SEM

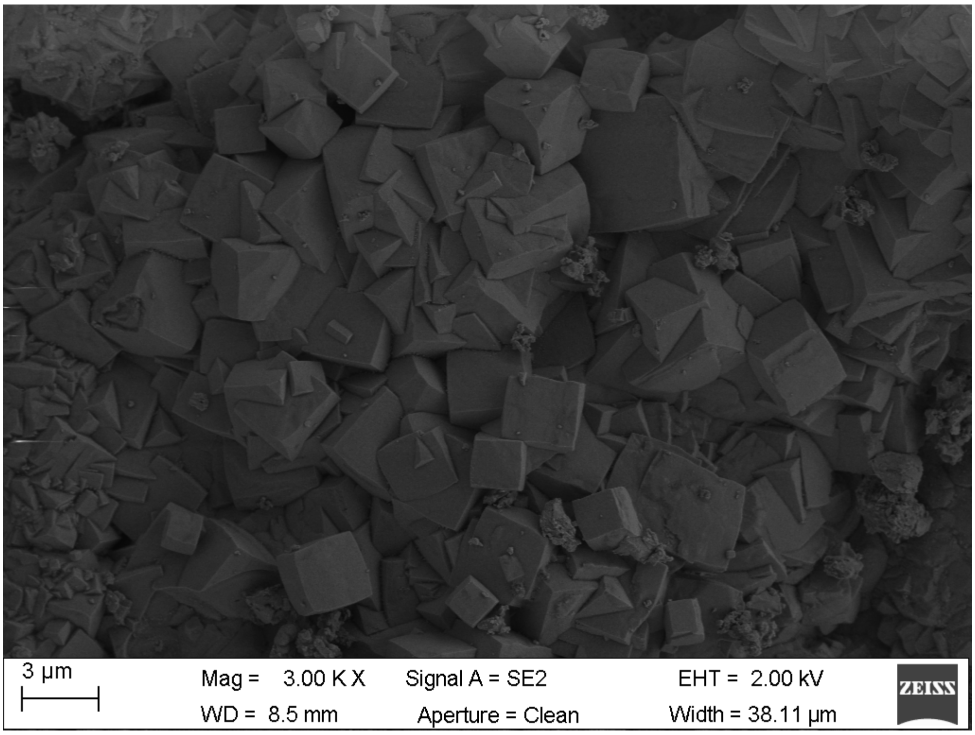

Figure S10 SEM image of $K_2Se_2C_2$.

4 DFT calculations

4.1 Solid State DFT calculations

All solid state DFT calculations were carried out using QUANTUM ESPRESSO 7.3 and its PWscf code.[6,7] Total energies and geometries were obtained using the PBEsol exchange functional[8], pseudopotentials from the SSSP PBEsol Precision v1.3.0 library[9–13] and D3 correction[14].

Table S7 Converged parameters for DFT calculations of $4\times4\times4$ supercells and cells with $P4/mmm$ symmetry of $A_2Se_2C_2$.

	$Na_2Se_2C_2$	$K_2Se_2C_2$	$Rb_2Se_2C_2$	$Cs_2Se_2C_2$
k-grid ($4\times4\times4$)	$3\times3\times3$	$3\times3\times3$	$3\times3\times3$	$3\times3\times3$
k-grid ($P4/mmm$)	$14\times14\times7$	$10\times10\times5$	$12\times12\times6$	$12\times12\times6$
$E_{\mathrm{cut,W}}$ (Ry)	150	100	60	80
$E_{\mathrm{cut},\rho}$ (Ry)	1200	800	480	640

For all structures, at first the size of the k-mesh was converged with respect to total energy and stress. Afterwards, the kinetic energy cutoff for wavefunctions ($E_{\mathrm{cut,W}}$) was converged as well. The kinetic energy cutoff for charge density and potential ($E_{\mathrm{cut},\rho}$) was fixed at $8E_{\mathrm{cut,W}}$. All converged values are given in Table S7.

For calculations of formation enthalpies, the total energies of the corresponding elements were subtracted. Crystal structure data of the elements was taken from the ICSD[15–21].

Spectroscopic data was calculated using the PHonon code and Density-Functional Perturbation Theory (DFPT).[22] As the long-range disorder was only expected to have a minor effect,[23] these calculations were carried out on a smaller cell for ease of use. Calculations used the local

density approximation (LDA)[24] functional with norm-conserving ONCVPSP v0.4.1 pseudopotentials[25] as this approach was demonstrated to perform well for DFPT calculations.[26]

4.2 Gas phase DFT calculations

All calculations were carried out using ORCA 6.1.0 and its built-in functions.[27–32] All energies and geometries were obtained by DFT calculations using the B3LYP functional[33–36] with D4 correction[37–39] and ma-def2-QZVPP[40,41] basis sets.

Starting geometries were created in Avogadro 2[42,43] and preliminary optimised using Open Babel[44]. Afterwards, the geometry was optimised on the described DFT level using the TIGHTOPT command. To compute the non-resonant Raman spectra, the polarisability was computed analytically, derivatives of the polarisability were calculated numerically.

5 Vibrational spectroscopy

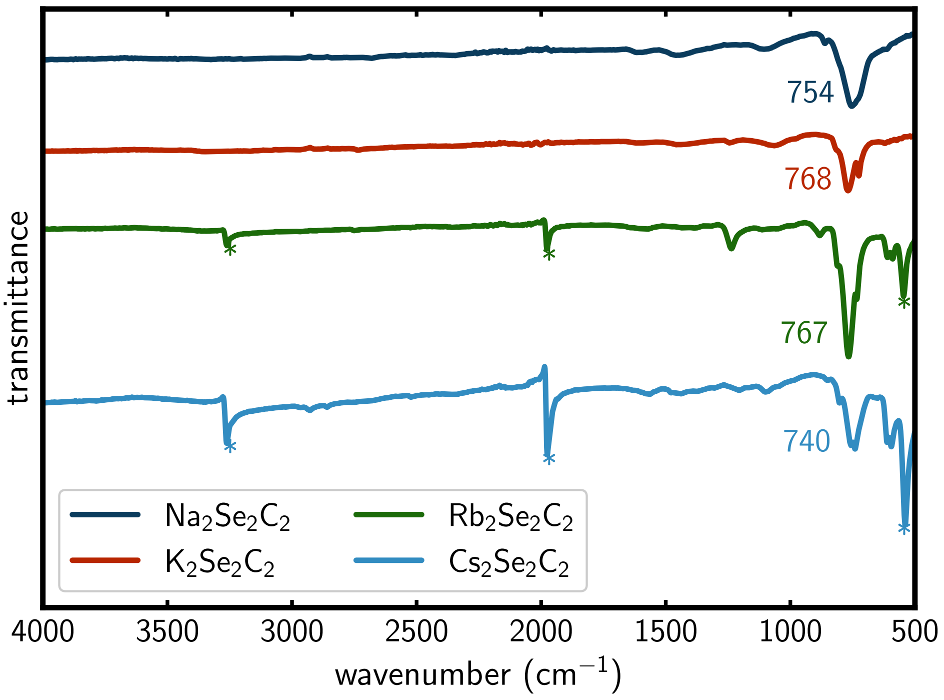

Figure S11 IR spectra of the $A_2Se_2C_2$ (A = Na–Cs) compounds. Wavenumbers of the Se—C stretching vibration are given. Modes that can be attributed to $RbSeC_2H$ or $CsSeC_2H$,[5] respectively, are marked with a *.

References

[1] D. A. Keen, *J. Appl. Crystallogr.*, 2001, **34**, 172–177.

[2] T. Proffen and S. J. L. Billinge, *J. Appl. Crystallogr.*, 1999, **32**, 572–575.

[3] P. Juhás, T. Davis, C. L. Farrow and S. J. L. Billinge, *J. Appl. Crystallogr.*, 2013, **46**, 560–566.

[4] L. Link and R. Niewa, *J. Appl. Crystallogr.*, 2023, **56**, 1855–1864.

[5] M. Hetzert, M. Werker and U. Ruschewitz, *Angew. Chem. Int. Ed.*, 2018, **57**, 16475–16479.

[6] P. Giannozzi, O. Andreussi, T. Brumme, O. Bunau, M. B. Nardelli, M. Calandra, R. Car, C. Cavazzoni, D. Ceresoli, M. Cococcioni, N. Colonna, I. Carnimeo, A. D. Corso, S. de Gironcoli, P. Delugas, R. A. D. Jr, A. Ferretti, A. Floris, G. Fratesi, G. Fugallo, R. Gebauer, U. Gerstmann, F. Giustino, T. Gorni, J. Jia, M. Kawamura, H.-Y. Ko, A. Kokalj, E. Küçükbenli, M. Lazzeri, M. Marsili, N. Marzari, F. Mauri, N. L. Nguyen, H.-V. Nguyen, A. O. de-la Roza, L. Paulatto, S. Poncé, D. Rocca, R. Sabatini, B. Santra, M. Schlipf, A. P. Seitsonen, A. Smogunov, I. Timrov, T. Thonhauser, P. Umari, N. Vast, X. Wu and S. Baroni, *J. Phys. Condens. Matter*, 2017, **29**, 465901.

[7] P. Giannozzi, S. Baroni, N. Bonini, M. Calandra, R. Car, C. Cavazzoni, D. Ceresoli, G. L. Chiarotti, M. Cococcioni, I. Dabo, A. Dal Corso, S. de Gironcoli, S. Fabris, G. Fratesi, R. Gebauer, U. Gerstmann, C. Gougoussis, A. Kokalj, M. Lazzeri, L. Martin-Samos, N. Marzari, F. Mauri, R. Mazzarello, S. Paolini, A. Pasquarello, L. Paulatto, C. Sbraccia, S. Scandolo, G. Sclauzero, A. P. Seitsonen, A. Smogunov, P. Umari and R. M. Wentzcovitch, *J. Phys. Condens. Matter*, 2009, **21**, 395502.

[8] J. P. Perdew, A. Ruzsinszky, G. I. Csonka, O. A. Vydrov, G. E. Scuseria, L. A. Constantin, X. Zhou and K. Burke, *Phys. Rev. Lett.*, 2008, **100**, 136406.

[9] G. Prandini, A. Marrazzo, I. E. Castelli, N. Mounet and N. Marzari, *npj Comput. Mater.*, 2018, **4**, 72.

[10] A. Dal Corso, *Comput. Mater. Sci.*, 2014, **95**, 337–350.

[11] M. Schlipf and F. Gygi, *Comput. Phys. Commun.*, 2015, **196**, 36–44.

[12] M. van Setten, M. Giantomassi, E. Bousquet, M. Verstraete, D. Hamann, X. Gonze and G.-M. Rignanese, *Comput. Phys. Commun.*, 2018, **226**, 39–54.

[13] K. F. Garrity, J. W. Bennett, K. M. Rabe and D. Vanderbilt, *Comput. Mater. Sci.*, 2014, **81**, 446–452.

[14] S. Grimme, J. Antony, S. Ehrlich and H. Krieg, *J. Chem. Phys.*, 2010, **132**, 154104.

[15] D. Zagorac, H. Müller, S. Ruehl, J. Zagorac and S. Rehme, *J. Appl. Crystallogr.*, 2019, **52**, 918–925.

[16] A. Ø. Drejer, M. S. Pedersen, M. Johansen and D. B. Ravnsbæk, *ACS Appl. Energy Mater.*, 2023, **6**, 4909–4921.

[17] R. Keller, W. B. Holzapfel and H. Schulz, *Phys. Rev. B*, 1977, **16**, 4404–4412.

[18] F. A. Kanda, R. M. Stevens and D. V. Keller, *J. Phys. Chem.*, 1965, **69**, 3867–3872.

[19] L.-G. Liu, *J. Phys. Chem. Solids*, 1986, **47**, 1067–1072.

[20] F. M. Kelly and W. B. Pearson, *Can. J. Phys.*, 1955, **33**, 17–74.

[21] C. E. Weir, G. J. Piermarini and S. Block, *J. Chem. Phys.*, 1971, **54**, 2768–2770.

[22] S. Baroni, S. de Gironcoli, A. Dal Corso and P. Giannozzi, *Rev. Mod. Phys.*, 2001, **73**, 515–562.

[23] G. Ulian and G. Valdrè, *Sci. Rep.*, 2023, **13**, 2725.

[24] P. Hohenberg and W. Kohn, *Phys. Rev.*, 1964, **136**, B864–B871.

[25] D. R. Hamann, *Phys. Rev. B*, 2013, **88**, 085117.

[26] L. He, F. Liu, G. Hautier, M. J. T. Oliveira, M. A. L. Marques, F. D. Vila, J. J. Rehr, G.-M. Rignanese and A. Zhou, *Phys. Rev. B*, 2014, **89**, 064305.

[27] F. Neese, *WIRES Comput. Molec. Sci.*, 2025, **15**, e70019.

[28] F. Neese, *J. Comput. Chem.*, 2003, **24**, 1740–1747.

[29] F. Neese, F. Wennmohs, A. Hansen and U. Becker, *Chem. Phys.*, 2009, **356**, 98–109.

[30] B. Helmich-Paris, B. de Souza, F. Neese and R. Izsák, *J. Chem. Phys.*, 2021, **155**, 104109.

[31] F. Neese, *J. Comput. Chem.*, 2022, **44**, 381–396.

[32] L. Wittmann, I. Gordiy, M. Friede, B. Helmich-Paris, S. Grimme, A. Hansen and M. Bursch, *Phys. Chem. Chem. Phys.*, 2024, **26**, 21379–21394.

[33] A. D. Becke, *J. Chem. Phys.*, 1993, **98**, 5648–5652.

[34] C. Lee, W. Yang and R. G. Parr, *Phys. Rev. B*, 1988, **37**, 785–789.

[35] S. H. Vosko, L. Wilk and M. Nusair, *Can. J. Phys.*, 1980, **58**, 1200–1211.

[36] P. J. Stephens, F. J. Devlin, C. F. Chabalowski and M. J. Frisch, *J. Phys. Chem.*, 1994, **98**, 11623–11627.

[37] E. Caldeweyher, C. Bannwarth and S. Grimme, *J. Chem. Phys.*, 2017, **147**, 034112.

[38] E. Caldeweyher, S. Ehlert, A. Hansen, H. Neugebauer, S. Spicher, C. Bannwarth and S. Grimme, *J. Chem. Phys.*, 2019, **150**, 154122.

[39] E. Caldeweyher, J.-M. Mewes, S. Ehlert and S. Grimme, *Phys. Chem. Chem. Phys.*, 2020, **22**, 8499–8512.

[40] F. Weigend and R. Ahlrichs, *Phys. Chem. Chem. Phys.*, 2005, **7**, 3297.

[41] J. Zheng, X. Xu and D. G. Truhlar, *Theor. Chem. Acc.*, 2010, **128**, 295–305.

[42] M. D. Hanwell, D. E. Curtis, D. C. Lonie, T. Vandermeersch, E. Zurek and G. R. Hutchison, *J. Cheminf.*, 2012, **4**, 17.

[43] K. Sharkey, M. D. Hanwell, C. Harris and A. Vacanti, *Avogadro 2 and Open Chemistry*, Online, 2013, `https://www.kitware.com/avogadro-2-and-open-chemistry/`, last visited 07.01.2026.

[44] N. M. O'Boyle, M. Banck, C. A. James, C. Morley, T. Vandermeersch and G. R. Hutchison, *J. Cheminf.*, 2011, **3**, 33.
